# Addressing health inequities in treating neuropathic pain: a scoping review of cognitive behavioral therapies, mindfulness, and meditation-based interventions

**DOI:** 10.1093/epirev/mxag002

**Published:** 2026-02-09

**Authors:** Robert Buren, Matteo Ponzano, Nathan T Adams, Jane Jun, Kathleen A Martin Ginis

**Affiliations:** School of Health and Exercise Sciences, University of British Columbia, Kelowna, British Columbia Canada; International Collaboration on Repair Discoveries, Blusson Spinal Cord Centre, University of British Columbia, Vancouver, British Columbia, Canada; School of Health and Exercise Sciences, University of British Columbia, Kelowna, British Columbia Canada; International Collaboration on Repair Discoveries, Blusson Spinal Cord Centre, University of British Columbia, Vancouver, British Columbia, Canada; School of Health and Exercise Sciences, University of British Columbia, Kelowna, British Columbia Canada; International Collaboration on Repair Discoveries, Blusson Spinal Cord Centre, University of British Columbia, Vancouver, British Columbia, Canada; Division of Physical Medicine and Rehabilitation, Department of Medicine, University of British Columbia, Vancouver, British Columbia, Canada; School of Health and Exercise Sciences, University of British Columbia, Kelowna, British Columbia Canada; International Collaboration on Repair Discoveries, Blusson Spinal Cord Centre, University of British Columbia, Vancouver, British Columbia, Canada; Division of Physical Medicine and Rehabilitation, Department of Medicine, University of British Columbia, Vancouver, British Columbia, Canada; Centre for Chronic Disease Prevention and Management, University of British Columbia, Kelowna, British Columbia, Canada

**Keywords:** neuropathic pain, health inequities, cognitive behavioral therapy, mindfulness, chronic pain

## Abstract

Chronic pain is the leading cause of disability worldwide and chronic neuropathic pain (NP) disproportionately affects individuals with unmet health care needs, especially those facing health inequities. This scoping review addresses 3 research questions: (1) What are the characteristics and outcomes of cognitive behavioral therapy (CBT), mindfulness, and meditation-based intervention studies designed to manage NP? (2) Do these intervention studies include participants from populations experiencing health inequities? (3) Are the interventions customized to meet the needs of people experiencing health inequities? Ten databases were searched for studies focused on the search terms “cognitive behavioral therapy,” “CBT,” “mindfulness meditation,” “and neuropathic pain.” In total, 1732 abstracts were screened and a total of 24 articles from 22 original studies were included. The primary characteristics of the studies showed that 99% of participants had NP attributable to diabetes, cancer, or spinal cord injury. Outcomes were consistent with previous reviews, demonstrating promise for CBT, mindfulness, and meditation-based therapies in relieving NP. However, demographics were poorly reported, and there was little diversity among participants. Individuals from groups experiencing health inequities were largely unrepresented. Although 55% of studies tailored intervention materials and provided content to support long-term practice, few included long-term follow-up to evaluate sustained impact. In general, research on this topic has fallen short in including and addressing the needs of people experiencing health inequities. Future research should prioritize broader participant inclusion criteria, involve individuals with lived experience in intervention design and delivery, and conduct long-term follow-up to enhance the accessibility, relevance, and sustainability of NP interventions.

## Introduction

Chronic pain is the leading cause of disability worldwide,^1^ accounting for more than 100 million years lived with disability. Neuropathic pain (NP) is a particularly severe form of chronic pain that affects up to 10% of the population.^2-5^ Neuropathic pain is defined as “pain arising as a direct consequence of a lesion or disease affecting the somatosensory system,”^6^ and described as burning, shooting, stabbing, squeezing, electric, or shock-like sensations.^7^ NP varies widely in etiology,^8^ often emerging secondarily to chronic conditions such as diabetes, cancer, stroke, and spinal cord injury (SCI).^9^

The epidemiology of NP is poorly understood^10^ but is known to disproportionately affect people experiencing health inequities. As defined by the World Health Organization, health inequities are avoidable differences in health outcomes or access to health care resources that stem from social, economic, or environmental disadvantages.^11^ These disparities reflect the unequal distribution of money, power, and resources within and between societies. Health inequities develop from a combination of social and structural factors, including education, income, gender identity, and disability status. Consistent with this definition, a review of population-level studies identified older age, female sex, inability to work, working in a manual-labor occupation, lower educational attainment, living in a rural area, and living in social housing as risk factors for NP.^12^^,^^13^ In the largest epidemiological study of NP to date (*n* = 148 828), NP was significantly associated with age, social deprivation, manual and personal service–type occupations, and worse health-related quality of life (HRQoL).^9^

People with NP report more severe pain than do people with pain that is not NP,^9^^,^^13^ partly because of a lack of effective treatments. The recommended first- and second-line NP treatments are pharmacological (eg, gabapentinoids, tricyclic antidepressants) and provide at least 50% pain relief for less than half of people treated.^14^ Given difficulties treating NP, multidisciplinary treatment approaches have been recommended, including psychologically based therapies.^15^

Cognitive behavioral therapy (CBT), mindfulness, and meditation-based therapies are the psychology-based interventions used most often to treat NP.^16-18^ The focuses of these interventions range from modifying patients’ thoughts, beliefs, and behavioral responses to pain,^19^ to fostering adaptive coping mechanisms and acceptance.^20^ Whereas traditional CBT approaches focus on altering pain-related cognitive processes and improving pain coping skills and self-management by addressing dysfunctional beliefs and thought patterns,^21^^,^^22^ current CBT approaches, such as acceptance and commitment therapy, and dialectical behavioral therapy, focus on emotional regulation and psychological flexibility.^23^ Mindfulness-based approaches emphasize awareness of thoughts and bodily sensations, enhanced self-regulation and reduced automatic responses, and observation of experiences without judgment.^24-26^ Finally, meditation-based interventions (eg, mindfulness yoga, Qigong) reduce stress and enhance self-awareness and well-being through focused movement combined with attention practice.^24^

Although a recent survey^27^ showed multidisciplinary pain management techniques (eg, meditation, mindfulness) are used widely, there is a lack of high-quality research on these interventions, especially for treating NP.^18^ A recent scoping review of psychology-based interventions for NP^18^ included only interventions delivered by qualified psychologists or trained psychological therapists, and only participants who had clinical assessments and diagnoses of NP. Although the review is informative, it may be biased toward studies of people facing fewer health inequities; that is, people with access to health care who can secure a clinical diagnosis and travel to an interventionist’s location.

Cognitive behavioral therapy, mindfulness, and meditation-based therapies may be low-barrier NP treatment options for large segments of the population, including those experiencing health inequities. These types of interventions have the potential to be implemented on a large scale relatively inexpensively.^28^ Yet, it is not clear whether researchers are testing these interventions in diverse samples and if or how interventions are being customized to address the specific needs of these groups. Given calls to make pain research more inclusive and to develop culturally appropriate pain treatments,^29^ these are important questions to answer as researchers work toward developing population-level approaches to managing NP.^10^ We undertook a review to address these questions.

## Methods

The scoping review process was guided by Arksey and O’Malley’s 5-stage scoping review methodological framework.^30^ The review team included researchers with expertise in pain and health-behavior change and promotion in populations with disabilities. The lead author (R.B.) is an individual with SCI who has experienced NP for 17 years and whose lived experience informed the research questions and interpretation of findings. The Prospective Register of Systematic Reviews does not allow for registration of scoping reviews. We also applied the Cochrane PROGRESS-Plus framework,^31^ which identifies the key equity dimensions of place of residence, race or ethnicity, occupation, sex/gender, religion, education, socioeconomic status, and social capital, along with additional factors such as age and disability. This framework helped structure our data assessment and identify how interventions addressed, or failed to address, the needs of individuals experiencing NP.

### Stage 1: identify the research question

We addressed 3 research questions: (1) What are the characteristics and outcomes of CBT, mindfulness, and meditation-based intervention studies designed to manage NP? (2) Do these intervention studies include participants from populations experiencing health inequities? (3) Are the interventions customized to meet the needs of people experiencing health inequities? These questions align with a key purpose of conducting a scoping review: to examine how research is conducted on a certain topic or field.^32^

### Stage 2: identify relevant studies

The literature search was performed by a medical librarian in October 2022 and updated in October 2023. Ten databases were searched: MEDLINE (Ovid), Psycinfo, PubMed, Cumulative Index to Nursing and Allied Health (CINAHL), Web of Science, Cochrane, and PROSPERO, as well as 3 gray literature databases: Trip, Canada Commons, and Google Scholar. We searched the databases from inception using variations of 4 core keyword search strings: cognitive behavioral therapy, CBT, mindfulness meditation, and neuropathic pain (Appendix S1). The search was restricted to English-language publications. Citations were exported into Covidence, where duplicates were automatically removed. Reference lists of retrieved studies were searched for additional relevant studies.

### Stage 3: study selection

Inclusion criteria were as follows: (1) studies involving individuals experiencing NP for at least 3 months; (2) participants 18 years of age or older; (3) delivered a CBT, mindfulness, or meditation-based intervention; and (4) administered any validated measure of pain (eg, pain intensity, pain interference) or pain-related outcomes (eg, pain acceptance, pain catastrophizing), henceforward referred to collectively as “pain measures.”

Exclusion criteria were (1) studies in which NP was reported by less than 50% of the sample (2) participants’ NP etiology was an infectious disease (eg, AIDS, shingles, COVID-19), because we wanted to focus on the leading causes of NP (ie, noninfectious disease)^33^; (3) articles that were not original research, such as systematic and scoping reviews, meta-analyses, letters to the editor, commentaries, conference presentations; and, (4) studies written in languages other than English, because a budget for translation was not available. Because we had research questions pertaining to health inequities, we included all research designs, allowing for the potential inclusion of underrepresented or heterogeneous samples that may be excluded in controlled trials. Unlike the Oguchi et al. review,^18^ we did not exclude studies in which participants self-identified as having NP, or when the intervention was not delivered by a psychological professional.

Two authors (R.B. and M.P.) independently screened titles and abstracts (level-1 screening) and full-text (level-2 screening) using Covidence. Discrepancies were resolved by discussion or a third author (N.A. or K.M.G.).^34^

### Stage 4: extracting and charting data

From the included articles, 2 authors (R.B. and M.P.) independently extracted information on the primary study characteristics, pain and HRQoL outcome measures and outcomes, and characteristics of the interventions delivered in the studies. Data extraction was performed using a standardized Microsoft Excel form developed by the research team to ensure clarity and consistency to facilitate the data-charting processes.

First, primary study characteristics were extracted, including author name(s), year of publication, country where the study was conducted, study design, measurement time points, participants’ etiology of NP, and participant demographics (sex/gender, age, marital and employment status, education, ethnicity, and race). Second, pain outcome measures and outcomes were extracted, including pre- and postintervention values and any statistically significant differences (*P* < .05). The frequency of use of each pain outcome measure was calculated. Because people with NP experience inequities in HRQoL,^9^ we also extracted HRQoL outcome measures and outcomes. HRQoL was defined as an individual’s or a group’s perceived physical and mental health over time^35^ and those components of QoL that are directly and indirectly affected by health, disease, disorder, and injury (eg, symptoms, physical, cognitive, emotional and social functioning).^36^^,^^37^ Outcomes were extracted that aligned with these definitions.

Third, basic intervention information was extracted, including intervention type (CBT, mindfulness, meditation-based), duration, and location of delivery (eg, clinic, home). To address questions about customization of interventions (including whether interventions addressed participants’ long-term needs for NP management or treatment), we also extracted the following information: whether content was modified during delivery to address participants’ needs; if individuals with lived experience were involved in intervention delivery; if participants were provided with media resources to practice the intervention on their own (eg, digital copies of a guided mindfulness meditation); and if the intervention incorporated strategies to facilitate and encourage continued practice beyond the study period.

To further explore whether barriers to nonpharmacologic therapies may differ by NP etiology, we conducted additional comparisons among diabetes, cancer, and SCI studies. Data were extracted by NP etiology to identify potential differences among the percentages of studies that reported statistically significant pain and HRQoL outcome measures, intervention delivery, tailoring, independence to self-administer, and follow-up duration. This step was added to strengthen our understanding of whether pain etiology itself may act as a condition contributing to health inequities.

Assessments of risk of bias and methodological quality are not typically included in scoping reviews.^38^ We did, however, assess the quality of the interventions using a warp and weft mindfulness-assessment tool, which was designed to code and evaluate the content and delivery of mindfulness-based interventions.^39^ “Warp” elements represent the core, unchanging intervention components essential for an intervention to be classified as mindfulness based (eg, psychological principles, evidence-based practices). “Weft” elements capture adaptable aspects of an intervention that can be modified to fit specific populations, contexts, or individual needs. Coding for warp and weft provides information on whether mindfulness-based interventions are both consistent in their foundational approach and sufficiently flexible to serve diverse communities effectively.

The tool consists of a 13-item checklist of intervention content and delivery components that are coded yes or no*.* We added 2 more codes: “assume yes” and “do not know.” We coded “assume yes” those elements not explicitly reported in the article being reviewed, but, based on other study details (eg, the use of a standardized intervention that, by definition, includes certain warp and weft elements), we were confident the elements were present. We coded “do not know” those elements for which it was unclear if they were delivered. In addition to looking at the overall pattern of warp and weft scores, 2 specific warp and weft items were used to address our health equity questions: the tailoring of the intervention to the study population or sample; and if the instructor had knowledge, experience, and professional training related to the study population or sample.

### Stage 5: collating, summarizing, and reporting results

Figures and tables were created to collate and summarize study information. Descriptive, summary statistics were calculated for study and intervention characteristics (ie, frequency [*n*] and percentages, and mean [SD]). Results are organized and presented according to the research questions.

## Results

### Primary study characteristics

**Figure 1:**
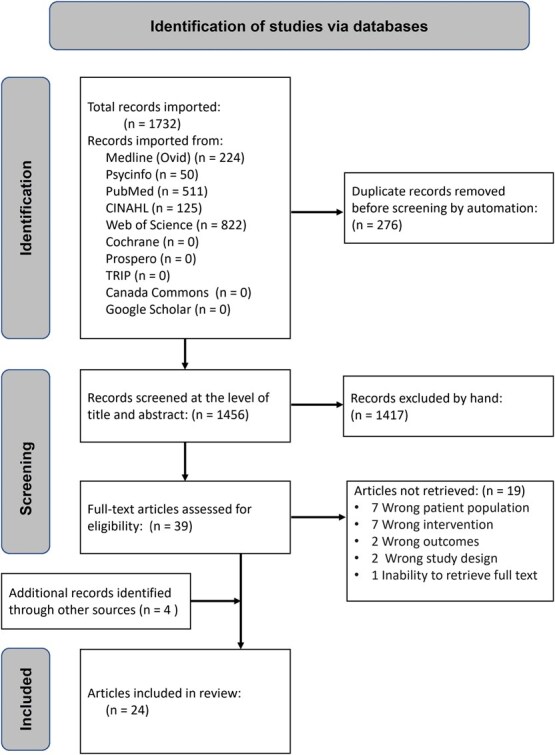
Preferred Reporting Items for Systematic Reviews and Meta-Analyses (PRISMA) flow diagram of study inclusion and exclusion.

The literature searches yielded a total of 1732 deduplicated titles and abstracts. Of these, 39 articles were identified for full-text screening. Checking reference lists of the retrieved studies led to the identification of 4 additional studies,^40-43^ resulting in a total of 24 articles, from 22 original studies (Figure 1). Table 1 provides primary study characteristics. Table 2 details etiology of NP. All 24 articles were published between 2006 and 2023. Studies were conducted in 10 countries: the United States (*n* = 7); Canada (*n* = 6); Iran (*n* = 2); and 1 each in Australia, Denmark, Ireland, the Netherlands, Pakistan, Sweden, and Turkey. A total of 15 studies were randomized controlled trials (RCTs)^40^^,^^41^^,^^43-55^; 2 were nonrandomized controlled trials,^56^^,^^57^ 4 were single-arm feasibility trials,^42^^,^^56–58^ and 1 was a case study.^2^ None of the studies was purely observational. Most of the participants (*n* = 720; 64%) were women. The reporting of demographic data was limited and inconsistent across the 22 studies (Table 3). Age and sex/gender were reported in all 22 studies, education was reported in 11 studies; employment status in 9 studies; race or ethnicity in 8 studies; relationship status in 9 studies; socioeconomic status in 3 studies; and religion in 1 study.

**Table 1:** Characteristics of studies included in the scoping review (*n* = 22)

**First author, year, country** ^a^	**Study design**	**Type**	**No.**	**Duration (weeks)**	**Injury or condition overall**	**Follow-up time frame (weeks)**	**Key findings**
Brown, 2017, Australia^2^	Case study	Mindfulness	1	12	Stroke	26	The participant had a clinically meaningful reduction in pain, emotional reactivity, depression, and stress.
Davoudi, 2021, Iran^47^	RCT	Mindfulness	225	12	Diabetes	12	Vitamin D supplementation and mindfulness training reduced pain severity and pain-related disability and improved QoL.
Hatchard, 2021, Canada^45^	RCT	Mindfulness (MBSR)	21	8	Breast cancer	10	Results suggest MBSR has a marked impact on how pain is processed, and for survivors of breast cancer it is a viable adjunctive treatment option.
Hussain, 2019, Pakistan^43^	RCT	Mindfulness	105	8	Diabetes	20	Benefits of mindfulness meditation can include reduction in pain-related medication consumption, better treatment outcomes, and improvement in comorbid conditions (eg, anxiety, depression).
Izgu, 2020, Turkey^50^	RCT	Mindfulness	77	12	Diabetes	14	Progressive muscle relaxation and mindfulness meditation reduced the severity of diabetic peripheral neuropathy.
Johannsen, 2016, Denmark^49^	RCT	Mindfulness (MBCT)	129	8	Breast cancer	32	MBCT showed a statistically significant, robust, and durable (6-month follow up) effect on pain intensity, indicating MBCT may be an efficacious pain rehabilitation strategy for women treated for breast cancer.
Mioduszewski, 2020, Canada^51^	RCT	Mindfulness (MBSR)	23	8	Breast cancer	8	MBSR training may enhance the integrity of cerebral white matter. Brief Pain Inventory scores for Ps and Pi were significantly decreased after the 8-week training program.
Nathan, 2017, Canada^40^	RCT	Mindfulness (MBSR)	62	8	Diabetes	20	Participation in an MBSR course improved function and reduced pain intensity, pain catastrophizing, depression, and perceived stress while improving HRQoL.
*Rozworska, 2020, Canada* ^59^^,^^60^	RCT	Mindfulness (MBSR)	62	8	Diabetes	20	The results suggest MBSR improves pain-related outcomes by increasing patients’ levels of mindfulness in line with the theorized mechanism of change in MBIs.
Shergill, 2022, Canada^54^	RCT	Mindfulness (MBSR)	98	8	Breast cancer	20	MBSR may not reduce pain-related disability or improve QoL among women with CNP after breast cancer more than did waiting for treatment (compared with waitlist control group).
Smith, 2021, Canada^52^	RCT	Mindfulness (MBSR)	23	8	Breast cancer	10	Changes in the brain (default mode network) after MBSR intervention are associated with alterations in experienced pain severity.
Teixeira, 2010, United States^41^	RCT	Mindfulness	20	4	Diabetes	4	No statistically significant improvements in QoL, pain, or sleep after the mindfulness meditation intervention.
Burke, 2019, Ireland^44^	RCT	CBT (PMP)	69	6	SCI	24	Reduction of pain intensity and Pi. Improvements were maintained at 3 months after the end of the intervention.
Heutink, 2012, the Netherlands^55^	RCT	CBT (custom)	61	10	SCI	34	A small, nonsignificant decrease in pain intensity was found. Significant treatment effects were found for anxiety and participation in activities, showing potential coping benefits of custom CBT.
*Heutink, 2014, the Netherlands* ^60^ ^,^ ^62^	RCT	CBT (custom)	29	10	SCI	66	Custom CBT may have long-lasting (12-month) improvements on pain intensity, pain-related disability, and anxiety; increased participation in activities.
Higgins, 2022, United States^53^	RCT	CBT	47	10	Diabetes	46	CBT as an adjunct treatment for patients with diabetic peripheral neuropathic pain may decrease the experience of pain intensity and improve mental health function.
Kioskli, 2020, United States^42^	Non-RCT	CBT (ACT)	30	5	Diabetes	17	Improvements in pain intensity, distress, depressive symptoms, functional impairment, cognitive fusion, committed action, self-perception, and pain acceptance.
Norrbrink Budh, 2006, Sweden^56^	Non-RCT	CBT	38	10	SCI	66	Reduced anxiety and depression symptoms, and tendency toward better quality of sleep and improved sense of coherence
Otis, 2013, United States^46^	RCT	CBT	19	11	Diabetes	27	CBT may reduce Ps and Pi associated with painful diabetic peripheral neuropathy.
Taheri, 2020, Iran^48^	RCT	CBT (ACT)	41	8	Diabetes	20	ACT can improve pain acceptance and reduce pain perception.
Carson, 2016, United States^58^	Non-RCT	Meditative: yoga	7	8	Fibromyalgia	8	Mindful yoga intervention may modulate abnormal pain processing in fibromyalgia, increasing physical strength, balance, and pain coping.
Galantino, 2020, United States^57^	Pilot study	Meditative (somatic yoga	8	8	Cancer	8	Somatic yoga and meditation may affect fear of falling and QoL in survivors of cancer who have CIPN.
Van de Winckel, 2023, United States^57^	Non-RCT	Meditative: Qigong	18	12	SCI	68	Remote Spring Forest Qigong’s “Five Element Qigong Healing Movements” practice is feasible in adults with SCI-related NP, with potential for NP relief and improvement in SCI related symptoms such as reduced spasm frequency and severity, and improved sleep.
Burns, 2013, Canada^61^	Non-RCT	Interdisciplinary program for chronic pain after SCI	22	10	SCI	68	Interdisciplinary pain program does not reduce Ps, but it can help persons with SCI and chronic NP cope with pain, lessen interference of pain, and improve their sense of control.

Abbreviations: ACT, acceptance and commitment therapy; CBT, cognitive behavioral therapy; CIPN, chemotherapy-induced peripheral neuropathy; HRQoL, health-related quality of life; MBCT, mindfulness-based cognitive therapy; MBI, mindfulness-based intervention; MBSR, mindfulness-based stress reduction; NP, neuropathic pain; Pi, pain interference; PMP, pain management program; Ps, pain severity; QoL, quality of life; RCT, randomized controlled trial; SCI, spinal cord injury.

a Italics denotes a second article from a previous study.

**Table 2:** Etiologies of neuropathic pain reported in articles included in this review

**Etiology**	**Studies (*n* = 22)**	**Participants (*n* = 1123)**
Diabetes	9	605
Cancer	6	302
Spinal cord injury	5	208
Fibromyalgia	1	7
Stroke	1	1

**Table 3:** Study Participant Demographics

**First author, year**	**Age** **(mean)**	**Sex/gender, no. (%)**	**Education level**	**Employment status**	**Race/ ethnicity**	**Relationship status**	**Income/SES**	**Religion**	**Location of residence**
Brown, 2017^2^	62	Female/woman, 1 (100)	2 years post-secondary	Volunteer part time, 3 d wk^−1^					
Burke, 2019^44^	51	Female, 17 (25) Male, 52 (75)		Not working/retired, 52% In employment training, 45% Not reported, 3%		Married /in a relationship, 71% Single/separated, 28% Not reported, 1%			
Burns, 2013^61^	48	Female, 6 (35) Male, 11 (65)	University, 38% College, 38% High school, 12% Less than high school, 12%	Employed, 24% Volunteer, 11% None/retired, 65%					
Carson, 2016^58^	49.7	Female, 7 (100)			Caucasian, 85.7% Native American, 14.3%	Married/partnered, 4 (57.1%) Divorced/separated, 2 (28.6%) Never married, 1 (14.3%)			
Davoudi, 2021^47^	54.8	Female, 92 (45) Male, 112 (55)							
Galantino, 2020^57^	65	Female, 7 (86) Male, 1 (14)			Black/, African American, 63% Caucasian, 37%				
Hatchard, 2021^45^	48.37	Female, 21 (100)			Caucasian, 90%				
Heutink, 2012^55^	58.8	Female, 22 (36) Male, 39 (64)	Low, 31% Middle, 41% High, 28%			Married/cohabitating, 84% Not cohabitating, 16%			
Heutink, 2014^60^^,^^62^	56.5	Female, 8 (18) Male, 21 (72)	Low, 48% Incomplete primary education, primary school, junior secondary technical education, general secondary education. (lower level)			Married/living with a spouse, 73%			
Higgins, 2022^53^	62.3	Female, 3 (6) Male, 44 (94)	>12 years, 70%		White, 70% Black, 26% Hispanic, 2% American Indian/Alaska Native, 2%	Married, 47%			
Hussain, 2019^43^	63.8	Female, 105 (100)	No education, 15% 1st to 11th grade, 48% High school graduate, 20% Vocational training, 14% College degree, 8%		Pakistani, 100%		Annual income of household, ≤$10 000, 95% $10 001-$20 000, 6% Did not answer, 4%		
Izgu, 2020^50^	61.6	Women, 46 (60) Men, 31 (40)	At least primary school, 70%			Married, 86%			
Johannsen, 2016^49^	56.7	Woman, 129 (100)	Lower (<2 years of FE), 38% Medium (2-4 years of FE), 50% Long (>5 years of FE), 10% Missing data, 2%	Full- or part-time, 40% Unemployed or receiving sickness benefit, 14% Retired, 40% Missing data, 6%		Married/cohabitating, 67% Single/not cohabitating, 33%			
Kioskli, 2020^42^	51.2	Female, 13 (43) Male, 17 (57)		Employed full-time, 30% Employed part-time, 10% Unemployed due to pain, 30% Unemployed, unrelated pain, 3% Student/training full-time, 3% Retired, 24%	White, 87% Asian, 7% Mixed race, 7%	Alone, 5 (16.7) With partner, 10 (33.3) With child/children, 2 (6.7) With partner and child/children, 8 (26.7) With other relatives, 3 (10) With friends/flatmates, 2 (6.6)			
Mioduszewski, 2020^51^	52.78	Female, 23 (100)							
Nathan, 2017^40^	59.7	Female/woman, 35 (56) Male/Man 27 (44)		Employed, 18% Retired, 53% Disability, 24% Other, 5%					
Norrbrink Budh, 2006^56^	53.2	Women, 24 (63) Men, 14 (37)							
Otis, 2013^46^	62.5	Male, 19 (100)	16.13 years of education	Retired, 75% Unemployed, 25%	Caucasian, 82%	Married, 38%			
Rozworska, 2020^59^^,^^60^		Female, 35 (56) Male, 27 (44)		Employed, 18%					
Shergill, 2022^54^	51.3	Female, 98 (100)		Employed full time, 34% Part time, 11% Unemployed, 13% Other, 42%	Caucasian, 82% African, 5% Asian, 2% First Nations, 2% Other, 4%				
Smith, 2021^52^	52.78	Female/woman, 23 (100)							
Taheri, 2020^48^	58.6	Female, 28 (68) Male, 13 (32)	Illiterate, 43% Less than a diploma, 43% Having diploma and higher, 15%						
Teixeira, 2010^41^	74.6	Female, 15 (75) Male, 5 (25)	High school graduate, 30% Some high school, 5% Some college courses, 25% College graduate, 15% Postgraduate, 25%		White, 90%African American/non-Hispanic, 5%Asian, 5%	Married, 35% Single, 5% Widowed, 50% Divorced, 10%	$0-$20 000, 20% $20 000-$40 000, 15% $40 000-$60 000, 25% ≥$80 000, 15% Do not wish to answer, 20% Missing data, 5%	Protestant, 35% Jewish, 35% Catholic, 15% Muslim, 5% Other, 10%	
Van de Winckel, 2023^57^	59.61	Female, 6 (33) Male, 12 (66) Othe,r 0 (0)			White, 100%		Socioeconomic distress, 67% Below the poverty threshold, 17%		Rural, 33% Urban, 67%

Abbreviations: FE, further education; SES, socioeconomic status. Note: Race/ethnicity as reported.

Education levels were variable across studies, ranging from 63% of participants with no education or less than high school^43^ to 100% of participants having college or postgraduate education.^58^ Full-time employment status ranged from 17% to 45%. Of the 8 studies that reported race or ethnicity, 6 studies consisted of predominantly (70%) or exclusively White participants. The remaining 2 studies had no White participants; 1 comprised Asian and Black participants,^56^ and the other was conducted with all Pakistani participants.^43^ Of the 9 studies that reported relationship status, the majority of participants were married or in a relationship (range across all studies, 43%-91%). In 2 of the 3 studies that reported socioeconomic status, most of the participants lived below the poverty threshold^63^ or in socioeconomic distress.^43^ In the third study, there was a relatively even dispersion of income levels, ranging from 15% to 25% across 4 income groups (from $0 to $20 000, to ≥$80 000).^41^ A mindfulness meditation study was the only study to report religion, with 5% of participants reporting to be Muslim, 10% other, 15% Catholic, 35% Protestant, and 35% Jewish.^41^ Across all studies, no baseline measures of disability were reported.^61^

### Pain and HRQoL outcomes and outcome measures

Nineteen studies (86%) reported at least 1 statistically significant (*P* < .05) improvement in a pain outcome. Of 80 statistical tests of changes or differences in pain, 45 (56%) were statistically significant. Thirteen studies (59%) included a postintervention follow-up of 3 months or longer, and 85% of these reported a statistically significant difference in pain relative to baseline. Four of these studies (18%) also conducted a 12-month follow-up, and 75% of these reported statistical significance relative to baseline. A total of 52 different pain measures were used.

Ten of the 17 studies (59%) that measured HRQoL reported a statistically significant (*P* < .05) improvement. Of 80 statistical tests of changes or differences in HRQoL, 33 (41%) were significant. Thirteen studies (76%) included a postintervention follow-up of 3 months or longer, and 92% of these reported a statistically significant difference in HRQoL relative to baseline. Four of these studies (31%) also conducted a 12-month follow-up, and 75% of these reported statistical significance relative to baseline. Sixty-two different HRQoL measures were used.

### Intervention characteristics

Four types of therapeutic interventions were delivered: mindfulness-based (*n* = 11), CBT (*n* = 7), meditative (*n* = 3), and multidisciplinary (*n* = 1) (Table 1). The duration of interventions ranged from 4 to 12 weeks (mean = 9.05; SD = 2.00). The majority (77%) of studies had interventions (namely, education and instruction) delivered weekly in a clinic or hospital setting. In the remaining studies (23%), the intervention was delivered remotely—either via portable digital media (eg, compact disc) or online—and participants could take part in a location of their choosing.

To address our second and third research questions, we examined whether individuals with disabilities contributed to intervention development and delivery, and whether the content was tailored to participants’ specific needs. Most interventions (91%) were delivered by a trained instructor or certified professional (eg, clinical psychologist, psychotherapist, certified mindfulness instructor), and 8 studies (37%) explicitly stated that the instructor had specialist knowledge related to the groups they were treating; however, none (0%) of the interventions was delivered by an individual with the same lived experience as the participants (eg, an individual dealing with diabetic neuropathy, an individual with SCI dealing with NP). In 55% of all interventions, the content was modified or tailored to the characteristics of the participants. For instance, in 1 intervention, yoga exercises were modified for participants with physical disabilities.^56^ In 2 studies that focused on people with SCI, there was explicit mention of incorporating descriptions and images of people with SCI in the intervention materials.^44^^,^^58^ Four interventions (18%) also modified the intervention during delivery, accommodating participant needs as they occurred (eg, changing posture or position, adding a supportive device like a pillow, changing the length of a pose).

We also looked at whether interventionists facilitated or encouraged practice of the intervention techniques beyond the study period. Twelve studies (55%) explicitly stated that knowledge or training was incorporated into the intervention with the goal of facilitating participants’ continued practice after the study was completed. Nine studies (41%) provided participants digital media that could be used to guide their practice after the study ended. Six studies (27%) incorporated specific intentional efforts (eg, having a buddy join the participant for at least 2 sessions) to facilitate and encourage continued practice beyond the study duration.

Beyond finding similar statistically significant improvements in pain outcomes across all etiologies—89% of diabetes, 83% of cancer, and 80% of SCI studies—notable differences were observed in the etiology-based comparisons. Significant improvements in HRQoL outcomes were less common, occurring in 33% of diabetes and cancer studies, and in none (0%) of the SCI studies. Tailoring of intervention delivery was most consistently described in SCI studies (100%), compared with 33% of diabetes and 50% of cancer studies. Although all studies were delivered by trained professionals, less than half of the intervention materials (40% SCI, 0% diabetes, 17% cancer) included individuals with lived experience, and none was delivered by a person with lived experience. The provision of intervention materials for self-administration was highest in SCI studies, with 100% providing these resources, compared with 11% of diabetes studies and 0% of cancer studies. Follow-up assessments of 3 months or longer were reported in all SCI studies, compared with 56% for diabetes studies and 33% for cancer studies. The only etiology group to conduct 12-month follow-up assessments was SCI (80%).

Regarding the overall quality and integrity of the interventions, across 286 fields (*n* = 22 interventions × 13 criteria) of warp and weft mindfulness assessment criteria, 41% were coded yes, 33% as assumed yes, 17% as do not know, and 9% as no (Table S1). Three studies^2^^,^^41^^,^^45^ had zero yes codes, indicating that none of the (warp and weft) mindfulness-related criteria were explicitly noted within the manuscript as being met.

## Discussion

People experiencing health inequities are at increased risk for chronic pain disorders, including NP.^9^^,^^12^^,^^13^ Cognitive behavioral therapy, mindfulness, and meditation-based interventions show promise for alleviating NP and have potential to be scaled up relatively inexpensively.^28^ This scoping review explored how research on these types of NP interventions is being conducted. We were particularly interested in whether the studies are inclusive of people facing health inequities and if the interventions are being designed and delivered in ways to foster inclusion and meet the needs of equity-owed groups.

### Question 1: What are the primary study characteristics and outcomes?

Our systematic search yielded 24 articles from 22 studies, the majority of which (77%) used a randomized or nonrandomized controlled trial design. Given that controlled trials are considered the strongest form of evidence for interventions,^64^ it is encouraging that most studies were of rigorous design. Scoping reviews can be used to determine whether there is sufficient evidence to undertake a subsequent meta-analysis^32^ to answer questions about a treatment’s efficacy and effectiveness. Our identification of 15 controlled studies of CBT, mindfulness, and meditation-based interventions suggests there may be sufficient controlled trials for a future meta-analysis to estimate the effects of these interventions on NP.

There are at least 45 different noninfectious disease–related causes of NP.^8^ However, 99% of participants in the reviewed studies were sampled from just 3 pain etiology groups: diabetes (48%), cancer (30%), or SCI (21%). The preponderance of studies focused on diabetes and cancer reflects the prevalence of these conditions worldwide^65^^,^^66^ and the pressing need to alleviate the global burden of disability associated with NP in these groups.^67^ In contrast, the number of people living with SCI worldwide (*n* = 20.6 million)^68^ is much smaller, but NP is 1 of the most frequent and debilitating consequences of SCI. Broadening research to encompass a wider range of pain etiologies could improve health equity by making all therapies more accessible to all individuals experiencing NP, regardless of the origin of their pain.

The vast majority of studies were conducted in high-income countries (77%).^69^ Just 3 studies were conducted in upper-middle-income countries (*n* = 2 in Iran and 1 in Turkey), and 1 in a lower-middle-income country (Pakistan). Although the prevalence of pain and disability is disproportionately greater in low- and middle-income countries, relatively little pain research is being done in these countries, because of insufficient training and limited funding.^29^

Scoping reviews are not meant to answer questions about intervention effectiveness.^32^ However, we considered it prudent to explore whether the findings based on studies included in this scoping review, which used somewhat broader inclusion criteria than previous reviews, were consistent with the findings of previous reviews. In 86% of the studies included in our review, the authors reported significant reductions in participants’ pain. These findings are consistent with previous reviews that focused on the effects of psychological interventions on diabetic peripheral neuropathy^70^ and psychology-based interventions for adults with chronic NP.^18^

The effects of the interventions were robust across a wide range of pain outcome measures. The number of different pain outcome measures (*n* = 52) used was striking, along with our observation that most measures (61%) were used in just a single study. The large number of measures may reflect that the reviewed studies captured 5 different etiologies of pain; researchers may favor outcome measures specific to the samples or etiologies they investigate. The wide variety of NP outcome measures highlights a key issue: without consistent outcome measures, it is difficult to make comparisons across studies and to draw conclusions about effectiveness across interventions, samples, and other study characteristics. Investigators wanting to conduct meta-analyses on these studies will need to address the challenge of inconsistent pain-outcome measures.

People living with NP experience disparities in HRQoL,^9^ so this is an important end point to consider when testing NP interventions. The majority of studies that measured HRQoL reported improvements (59%). A large range of measures was used (*n* = 62), with 78% used in just a single study. As with pain measures, the lack of consistency in measurement creates challenges for comparing outcomes across studies.

The longest intervention period was 12 weeks (range, 4-12 weeks). Few studies included a follow-up assessment. The relatively short duration of studies and the lack of long-term follow-up suggests greater focus on testing the efficacy of interventions for NP relief (ie, treating the symptoms of NP) rather than seeking a long-term cure for NP (ie, treating the underlying cause). This highlights a paradox and critical need. When researchers seek NP solutions but fail to measure long-term effects, research impact is undermined. A sufficient duration of testing is essential with a shift in focus from symptom management to cure. Without dedicated efforts to test for a cure, one is unlikely to be found.

### Question 2: Do the studies include participants from groups experiencing inequities?

In general, participants’ demographic characteristics were poorly reported. All studies reported participants’ age and sex/gender, and half reported education level, but many reported little to no additional demographics. Based on the available data, the study samples were largely middle-aged or older and included more female/women than male/men participants (*n* = 4 studies were conducted with female participants who had breast cancer and NP). None of the studies included people identifying with other genders. From an age and gender standpoint, the study samples included groups most at risk for NP. Across studies that reported relationship status, the majority of participants were in a partnered relationship. This information provides a crude indicator that most participants had at least some social support. Because people experiencing NP often report social isolation,^71^ the study samples may be biased toward individuals who benefit from this key social determinant of health.^72^

In studies that reported race/ethnicity, most participants identified as White/Caucasian. There were 2 exceptions; in the Galantino et al^56^ study of a somatic yoga and mindfulness intervention for patients with cancer, 63% of participants were African American; in the Hussein et al.^43^ study of a mindfulness intervention for a disenfranchised group of elderly women living in Pakistan with diabetic neuropathy, 100% of participants were Pakistani. Regarding indicators of socioeconomic status, when employment was reported, most participants reported having part- or full-time employment or being retired. Among the 3 studies that reported data on income, 1 study^43^ targeting disadvantaged elderly Pakistani women reported 95% of participants earning an annual income of $10 000 or less, and another,^63^ using volunteer sampling, found 84% of participants with SCI were either below the poverty threshold or in socioeconomic distress. Education levels were mixed. Samples ranged from predominantly low levels of education (less than high school) to exclusively high levels (college or graduate degrees).

None of the studies included an explicit measure of disability—that is, the extent to which participants experience difficulties, limitations, or challenges in performing and participating in normal daily activities.^73^ Collecting and presenting data on participants’ disability status are not only important for understanding who is or is not included in intervention research but also for understanding who is most likely to benefit from NP interventions.

Overall, the lack of demographic data makes it difficult to draw firm conclusions about the extent to which the studies are targeting participants from groups experiencing health inequities. Though 2 peripheral neuropathy studies targeted specific groups (elderly Pakistani women,^43^ and those between the ages of 50 and 92 years^41^), in most studies, the sample selection seems to have been driven more by convenience and less by efforts to include diverse representation. Although NP disproportionately affects non-White adults and people experiencing social deprivation,^74^ based on demographic data provided for ethnicity, study country/location, employment, and relationship status, most studies included privileged participants who are least marginalized.

### Question 3: Are interventions being customized to the needs of people experiencing inequities?

Traditionally, CBT, mindfulness, and meditation-based pain interventions have been delivered in person, by a therapist, in group settings. However, remotely delivered interventions have become increasingly common. Where a pain intervention is delivered can influence barriers to access and either alleviate or contribute to health inequities.^44^^,^^63^

In the reviewed studies, most interventions (77%) were delivered at a hospital or clinic. Although researchers may find it convenient and practical to conduct NP interventions at their hospital or clinic workplaces, the participants’ resources (financial, logistical, physical, and emotional) required to make regular trips to these research settings can be costly and exclusionary. Furthermore, many clinical research settings are not easily accessible to people with disabilities, creating further barriers to participation among those who might benefit the most.^75^ For people who can participate, the logistics of commuting to research locations may increase stress and pain.^76^^,^^77^

Barriers to access can be reduced when interventions are delivered remotely, thereby allowing people to participate from the comfort of their homes or other location of their choosing. In 5 of the reviewed studies (23%), the intervention was delivered remotely. Participants were provided an assortment of media resources to guide them through the intervention. Of note, statistically significant improvements were reported in all but 1^51^ of these studies. That 1 study’s intervention was a compact disc of guided meditations to be practiced over 4 weeks, the shortest intervention duration across all 22 reviewed studies and markedly shorter than the 9-week average. The intervention content, lack of specific instruction in mindfulness, and short duration may explain the null findings. Although more research is needed to assess the effectiveness of remotely delivered, psychology-based interventions for NP, our review shows this delivery approach can produce positive effects. As long as remote delivery does not create new barriers (eg, require that participants can afford and have access to computers or internet), it can improve access to research participation and treatment.

The tailoring of interventions is an essential feature for addressing the needs of people experiencing health inequities.^29^ About half of the reviewed studies (55%) tailored intervention content, meeting spaces, or duration of instruction to participant characteristics (eg, accommodating for age^41^ and decreased energy levels caused by NP).^53^ Modification of interventions during delivery occurred in 18% of studies. In 1 of the meditation-based interventions,^49^ for example, participants were encouraged to adapt their posture and position to meet their unique needs and abilities. This is an important adaptation because sitting still for long periods can aggravate NP.^78^ Likewise, in the Qigong study,^63^ the interventionists emphasized that practicing while lying down was just as acceptable as practicing while sitting or standing.

Participant or patient engagement in research design is key to ensuring that the values, needs, and preferences of research users are accounted for.^79^ Only 3 studies incorporated the perspectives of people with lived experience in their intervention materials.^56^^,^^58^^,^^80^ None of the reviewed studies involved interventionists or facilitators with lived experience of NP. Involving more relatable interventionists or facilitators could enhance participant motivation, engagement, and outcomes by demonstrating greater sensitivity to participants’ unique needs.

If CBT, mindfulness, and meditation-based interventions are ultimately to be scaled up and used to treat NP across populations, then users must be able to self-manage and sustain their practice. About half of the studies (55%) provided specific training to support ongoing CBT, mindfulness, or meditation-based practice after the study intervention, but less than half (41%) provided media to guide independent practice (eg, CDs, print materials). These findings suggest a myopic view, with researchers focused primarily on short-term testing and outcomes rather than ensuring materials and procedures for NP reduction are optimized for long-term adoption and potential long-term benefit. This perspective is further supported by the lack of studies with long-term (ie, 12 months or longer) follow-up.

When comparing across NP etiologies, notable differences emerged in how interventions were tailored and delivered. Studies targeting SCI-related NP stood out for their accessibility and longer-term engagement. These findings suggest etiology may shape both the design and delivery of interventions and may, itself, represent a factor that contributes to health inequity. Although it can be contentious to imply that 1 etiology is inherently more severe than another, we propose that individuals with SCI are more likely than those with diabetes or cancer to experience prohibitive mobility restrictions—a key dimension of disability.^81^ Encouragingly, SCI-focused researchers appeared more attentive to adapting and delivering interventions to meet participants’ functional needs. This finding suggests that although etiological differences exist and may contribute to health inequities, mobility limitations need not result in less accessible or less inclusive interventions. Furthermore, intervention effectiveness appeared comparable across etiologies (80%-89% reported statistically significant pain reduction). This finding suggests that when researchers appropriately tailor intervention content and delivery to participants’ needs, equitable access to and quality of NP interventions can be achieved, even among populations with varying levels of mobility and disability.

The warp and weft framework provided data to assess quality and integrity of the interventions. Overall, most studies delivered and reported foundational mindfulness components (ie, the warp components). In contrast, extraction of the weft components indicated significant gaps in tailoring and modifying interventions to suit their samples. The limited evidence of tailoring raises concerns about whether cultural, social, and contextual factors are being adequately considered. It is also concerning that few studies provided evidence that instructors were knowledgeable about the groups receiving the interventions.

Collectively, the studies suggest CBT, mindfulness, and meditation-based interventions, including those delivered virtually, have the potential to be designed for customization, self-administration, and sustainability. These interventions may offer accessible tools and resources that support equitable pain management, particularly for underserved groups, when purposefully developed with long-term adoption in mind. However, due to the nature of scoping reviews, definitive conclusions about the feasibility, effectiveness, or equity of these interventions cannot be made. The expectation of postintervention independence and the inclusion of individuals with lived experience to deliver or codeliver training are areas that warrant further exploration and could play an important role in the ongoing use of NP treatment methodologies.

### Recommendations for future research

Scoping reviews^32^ examine how research is conducted on a particular topic. Our review has provided a comprehensive description and comparison of research testing CBT, mindfulness, and meditation-based interventions for the treatment of NP. Our results provide the foundation for making 5 recommendations for future research.^32^

First, we strongly recommend researchers collect detailed demographic data from study participants, particularly information to assess and address health inequities. The Cochrane PROGRESS-Plus tool provides a framework for identifying demographic dimensions that can contribute to health inequities.^31^ It can also be used to inform and guide the selection of a study’s demographic measures and to support the design of more inclusive and equitable intervention studies.

Second, although pain is a significant contributor to global disability,^9^^,^^82^ none of the reviewed studies included a measure of disability in their demographics. Going forward, it is crucial to measure disability to build a better understanding of how NP contributes to the global burden of disability^10^ and how NP interventions might alleviate that burden. Examples of disability measures include the 6-item Washington Group Short Set on Functioning^83^ and the World Health Organization Disability Assessment Schedule.^61^

Third, researchers need to agree on a consistent measure of NP to use in intervention studies. Without common measures, it is difficult to compare results across studies and intervention types. The measure should be able to be administered remotely to alleviate burden on participants to travel to a research site. Examples of NP-specific measures that can be self- or interviewer-administered are the Douleur Neuropathique 4 Questionnaire^84^ and the Neuropathic Pain Symptom Inventory.^85^ We also encourage scientists to include follow-up assessments of NP as many months postintervention as possible. Follow-up data are needed to assess the long-term effects, feasibility, and maintenance of interventions.

Fourth, the limited involvement of individuals with lived experience in intervention design, delivery, and evaluation, is a serious gap in NP research. One way to address this is through community-based participatory research. Community-based participatory research engages community members as co-researchers, making interventions more relevant, culturally sensitive, and responsive to participants’ needs. Community-based participatory research methods can promote long-term sustainability by incorporating the insights of people with lived experience into strategies that alleviate barriers and support continued practice beyond the study period.^86^ These methods also have been identified as key to the successful scale-up of interventions for communities and populations. As such, it may be an especially important approach to foster equity for individuals with limited health care access.

Fifth, we encourage researchers to consider including people with different etiologies of NP in the same study. Although we appreciate this approach would require researchers to create different tailored intervention resources for each etiology group within a single study, greater inclusion would help reduce health inequities. Researchers who study pain etiologies with lower incidence rates (eg, SCI) would also benefit from studies with larger samples and greater statistical power.

### Strengths and limitations of the review

This scoping review has several strengths that extend knowledge generated in previous systematic and scoping reviews of psychological interventions for NP.^16^^‑^^18^^,^^70^ One strength is the inclusion of studies using different types of CBT, mindfulness, and meditation-based therapies and studies sampling participants with a range of NP etiologies. This approach provides a broader perspective on the types of interventions being used for NP management for people with a wider range of disabilities and other health conditions. A second strength is our comprehensive extraction and comparison of intervention content and delivery components and evaluation of the quality of the interventions using the warp and weft tool. As far as we are aware, previous scoping reviews have not provided such detailed information or intervention protocol evaluation. This information is important for assessing the accessibility of the interventions and their potential for advancing health equity, particularly for people with disabilities and others who have difficulty accessing health services. Related to this point, a third strength is our inclusion of research questions pertaining to health (in)equities. We have described the studies in ways that prompt consideration of and identify gaps in equity, diversity, and inclusion (eg, participant characteristics, intervention development and delivery). For researchers interested in scaling up and delivering psychology-based interventions on a population-wide basis, we have highlighted knowledge gaps that must be addressed regarding the design, delivery, and evaluation of interventions for people experiencing health inequities.

There are also some limitations. By restricting the review to English-language publications, we have missed studies conducted and published in other languages, potentially narrowing the geographic, ethnic, and socioeconomic diversity represented across study samples. Nevertheless, our findings provide valuable insight on research conducted in English-speaking countries and by English-speaking research groups. Additionally, we excluded studies in which the NP was caused by infectious diseases, which represent less than 10% of NP causes.^8^^,^^33^ And finally, our review is limited to the information provided by authors in their publications. Although our extraction of information with the warp and weft tool revealed many reporting gaps, it is possible that intervention features coded as “do not know” were actually included in the interventions but not reported.

## Conclusion

Overall, this scoping review provides a comprehensive synthesis of CBT, mindfulness, and meditation-based interventions for NP management with a focus on health equity. The primary characteristics of the studies showed that 99% of participants had NP attributable to diabetes, cancer, or SCI. Outcomes were consistent with previous reviews, demonstrating promise for CBT, mindfulness, and meditation-based therapies in relieving NP. However, demographics were poorly reported and, with little diversity among participants, individuals from groups experiencing health inequities were largely unrepresented. Although 55% of studies tailored intervention materials and provided content to support long-term practice, few included long-term follow-up. Although the increasing use of online interventions is a positive step, in general, previous interventions have fallen short of effectively addressing inequities in access to pain treatments and research. Future research should prioritize broader participant inclusion criteria, involve individuals with lived experience in design and delivery, and adopt long-term follow-up to enhance the accessibility, relevance, and sustainability of NP interventions.

## Supplementary Material

Web_Material_mxag002

## Data Availability

No new data were generated or analyzed in this study.
